# Exploring the Efficacy of Musa Cavendish Stem Extract (Mucase) as a Novel Wound Dressing: A Comparative Study With Sofratulle®

**DOI:** 10.7759/cureus.54411

**Published:** 2024-02-18

**Authors:** Nuraini K Amanah, Sugeng Mashudi, Siti Munawaroh, Auliya W Azzarin, Fadhilah N Karimah, Fahmie Gunawan

**Affiliations:** 1 Health Sciences, Universitas Muhammadiyah Ponorogo, Ponorogo, IDN; 2 Pharmacology and Therapeutics, Trade Business of Citra Alam Pharmacy Laboratory, Ponorogo, IDN

**Keywords:** rattus novergicus wistar, musa cavendish stem extract, sofratulle®, wound dressing, ­wound healing

## Abstract

Background

This investigation explores the wound-healing potential of Musa Cavendish banana components. Specifically, the Musa Cavendish stem extract (MUCASE), comparatively assessing its efficacy against the commercial conventional wound dressing Sofratulle® as a sterile gauze containing the antibiotic framycetin sulfate BP 1%, designed for accelerating wound healing. While Musa Cavendish banana components have been acknowledged for their topical applications, scarce research has scrutinized the specific impact of MUCASE as a wound dressing, especially concerning its comparison with Sofratulle®.

Purpose

The primary objective is to evaluate and compare the effectiveness of Sofratulle® and varied concentrations of MUCASE in managing incision wounds.

Materials and methods

Fifteen male *Rattus norvegicus* rats were randomly allocated into five groups, each subjected to distinct treatments: 40%, 20%, 10% MUCASE, Sofratulle®, and negative control. Over a seven-day treatment span, measurements of the exudation along with the incision wounds' surface area and the rate of wound contraction were conducted.

Result

The findings revealed significant differences in wound conditions within each group pre- and post-dressing application, except for the negative control and MUCASE 10% groups. Particularly, MUCASE 10% exhibited suboptimal outcomes compared to MUCASE 40%, 20%, and Sofratulle®, showcasing a non-significant ratio of wound healing (p > 0.05). A comparable potential was exhibited by MUCASE 40%, 20%, and Sofratulle® in accelerating the healing of incisional wounds.

Conclusion

Both Sofratulle® and MUCASE are deemed suitable as wound dressings to facilitate efficient and swift wound healing. Nevertheless, the study's outcomes suggest that MUCASE surpasses Sofratulle® in accelerating the healing process of wounds.

## Introduction

The complex wound-healing process has a series of clinical stages that necessitate appropriate wound management. The hemostasis-inflammation stage is a barrier against infection with fibrin clot forms and fluid loss, while the proliferation and remodeling/maturational stage facilitate tissue strength and volume recovery. Factors such as wound form, hypoxia, bacterial colonization, ischemia, reperfusion injury, alterations in cellular response, and defects in collagen synthesis impact the duration of this process [1,2]. Effective wound healing application strategies are deemed necessary to enhance positive clinical outcomes [3].

Meanwhile, the exploration of innovative approaches in wound healing applications, particularly those involving plant extracts as dressings, is ongoing. Research suggests that natural compounds derived from plant extracts possess antioxidant properties, neutralizing reactive oxygen radicals and inhibiting lipid peroxidation [4-6]. These compounds hold the potential to accelerate the process of wound healing. For instance, the proven effectiveness of biomass extract from Musa Cavendish banana (MACE) stem in accelerating wound healing and its use as a food supplement and stabilizing agent in the food industry has been demonstrated [7]. Despite this, the considerable surplus biomass produced from banana stems, leaves, and peels, particularly in East Java Province, Indonesia, presents a challenge [8,9]. Thus, utilizing Musa Cavendish biomass potentially enhances innovative strategies in the health sector.

The literature on herbal medicine acknowledges the numerous benefits of extracts from Musa Cavendish, including its ability to treat diabetes and its anti-inflammatory, antioxidant, antifungal, and antibacterial qualities [10]. Studies by Matook et al. confirm the significant influence of Cavendish banana peel extract as an antibacterial and antioxidant agent [11]. Additionally, research by Zhang et al. also suggests using raw banana stem nanocellulose in wound dressings [12]. Despite these promising attributes, more information is needed about the type of extract from Musa Cavendish banana stem (MUCASE) that can accelerate wound healing, as it has yet to be thoroughly examined as a wound dressing. Therefore, further study is required to fully comprehend MUCASE's potential as a wound dressing and its impact on the rate at which wounds heal.

Furthermore, this study assesses the efficacy of different concentrations of Musa Cavendish stem extract wound dressings. It compares them to commercial conventional wound dressings like Sofratulle®. Sofratulle is a sterile gauze containing the antibiotic framycetin sulfate BP 1%, demonstrating encouraging outcomes in wound healing [13]. Sofratulle® has been recognized for its ability to create and maintain a moist wound environment, a crucial factor in promoting optimal conditions for wound healing. Despite this, Sofratulle® presents challenges when dressing wounds, such as quick, uneven, and strong adherence to the attached gauze, accompanied by limited suppurative bleeding and infection, a worsening of the inflammatory response, and trauma that is highly hyperpigmented and slightly hypertrophic [14]. The long-term use of aminoglycoside antibiotics in Sofratulle® raises concerns about negative impacts, including developing other infections, such as fungal infections [15].

Consequently, the primary research question guiding this investigation is whether the application of MUCASE accelerates the wound healing process compared to conventional dressings. Additionally, this study explores the mechanisms MUCASE may contribute to enhanced wound healing outcomes. Therefore, this study aims to extract MUCASE and evaluate its efficacy as a natural wound dressing. The gel extracted from the stem will be tested in vivo to compare its performance with the commercial product Sofratulle®. This research is significant as it marks the first application of an extract from the stem of Musa Cavendish to wound dressings to accelerate the healing process.

## Materials and methods

Materials

The study was conducted at two institutes, Trade Business of Citra Alam Pharmacy Laboratory, Ponorogo, East Java, Indonesia, and Akafarma Sunan Giri Laboratory, Ponorogo, East Java, Indonesia. The study gathered fresh Musa Cavendish plant stems from Kunti and Pulung villages in Ponorogo, East Java, Indonesia. Utilizing the experimental observations in this study, an in vivo random control group design involving *Rattus norvegicus* rats was employed. Comparisons were made with the conventional wound dressing Sofratulle®. Ethical approval for the research was secured from the Institute of Health Science Ethical Clearance Committee in Strada Indonesia, Kediri, Indonesia, with reference number 3946/KEPK/X/2023. 

**Figure 1:**
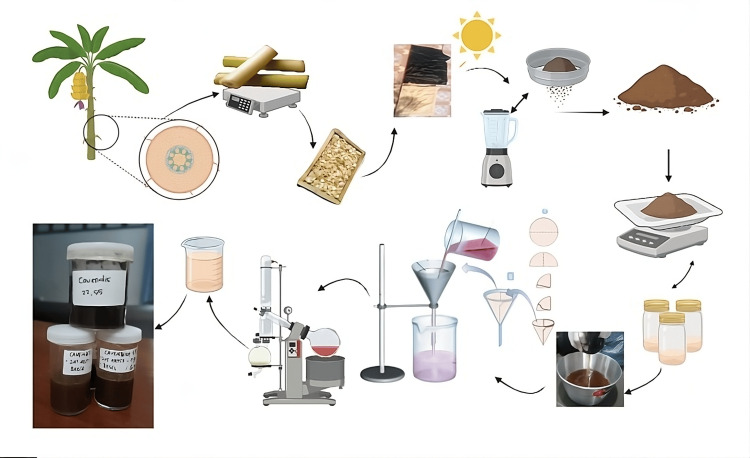
Preparation of crude material a: banana stems separated; b: weigh the raw stems; c: cut into squares and washed; d: drying process covered by black fabric; e: grinding and sifting process; f: banana stems powder; g: weighting and mixing with 70% alcohol and soaking for 24 hours; h: filtration process; i: evaporation process; j: extraction process added by hydroxypropyl methylcellulose (HPMC); k: extracted gel Image credits: Nuraini Khoirotun Amanah, created in Biorender.com

Following this, Musa Cavendish stems were cleaned and cut into thin pieces. Two processing methods were employed: oven-drying for six hours at 60 °C for the initial trial and indirect sun-drying for two to three days, which proved to be the best method after evaluation of the initial trial (Figure 1 for the extraction process). The processed stems were ground and sifted to produce a powder with a mesh size of 40 [16].

Extraction procedure

The maceration method of extraction involved placing 100 grams of Musa Cavendish stem powder into a container. Maceration was used to minimize thermal degradation and preserve the structural integrity of heat-sensitive MUCASE compounds. Maceration's cold extraction process, conducted at room temperature with a menstruum of 1000 ml 70% ethanol, was poured onto the material to ensure comprehensive coverage of the entire plant material. The container was tightly closed and left undisturbed for at least three days. The mixture was stirred periodically during this period, and the bottle was shaken occasionally to ensure even extraction. After the extraction, the miscella was separated from the remaining plant material through filtration or decantation. The resulting macerate was then filtered using filter paper, and the separation between the extract and the solvent was done through evaporation, which could be carried out using an oven or a water bath, resulting in a concentrated extract with a thick and dark brown consistency [16-18]. The filtration ensures satisfactory extraction yields while mitigating the risk of thermal degradation and addressing concerns related to the potential alteration of bioactive components.

The next step involved adding methylparaben and propylparaben as preservatives that had previously been dissolved in polyethylene glycol as a surfactant, cleanser, emulsifier, and humectant. Following the best possible homogeneity of the extract, the mixture was added to the hydroxypropyl methylcellulose (HPMC) solution and agitated until a homogenous consistency was produced. The combination was then sealed tightly and placed inside a tube.

Creating gel formulation and wound dressing MUCASE

In creating gel formulations, three variants containing Musa Cavendish stem extract were developed for wound dressings, with concentrations of 10%, 20%, and 40%. Each formulation contained 40 grams of the extracted gel, designed for twice-daily use over seven days. Gel compositions were structured based on Table 1 specifications. Gel-making involves weighing ingredients, mixing water with hydroxypropyl methylcellulose (HPMC), adding preservatives, and sealing the resulting mixture in a tube [19].

**Table 1:** Composition of the gel formulation derived from the stem extract of Musa Cavendish *: Gel matrix, F1: Gel containing 10% MUCASE, F2: Gel containing 20% MUCASE, F3: Gel containing 40% MUCASE HPMC: hydroxypropyl methylcellulose

No	Ingredient	F1 (10%) (g)	F2 (20%) (g)	F3 (40%) (g)
1	Banana stem extract	4 gr	8 gr	16 gr
2	Hpmc *	2 gr	2 gr	2 gr
	Polyethylene glycol *	6 gr	6 gr	6 gr
	Methylparaben *	0,16 gr	0,16 gr	0,16 gr
	Propylparaben *	0,16 gr	0,16 gr	0,16 gr
	Aqua ad	200 ml	200 ml	200 ml

**Figure 2:**
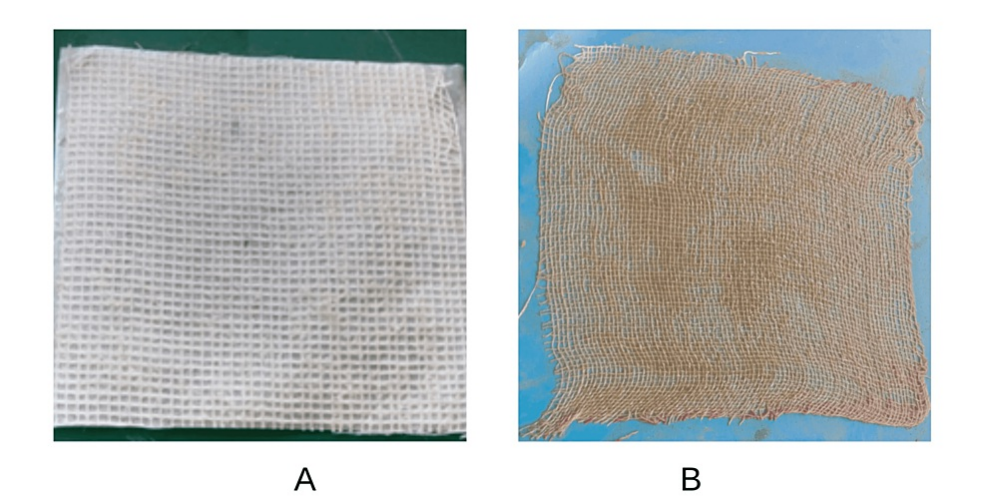
Comparison a: Sofratulle® dessing; b: MUCASE dressing

Creating dual wound dressings with dimensions of 10 cm x 10 cm from the gel formulation included soaking sterile gauze in a chitosan solution for 24 hours as a natural polymer for supporting antimicrobial efficacy and promoting mild wound healing. Dry it at 60-65°C for an hour to remove excess water, before creating dual wound dressings from the Musa Cavendish extract gel formulation, and apply 2 grams of gel (Figure 2 for comparison). The gauze was then sterilized using an autoclave. The remaining extract has an average of 1 gram after direct wound application around once daily, used over seven days to optimize and ensure the consistency of treatments. The positive results suggest that the wound may have absorbed some effective MUCASE extracts or experienced other processes during the application.

Surgical procedure on animals and evaluation of wound healing activity parameters

For the animal study, fifteen male Wistar rats (*Rattus norvegicus*) were procured from Akafarma Sunan Giri Laboratory, Ponorogo, East Java, Indonesia. The rats weighed between 140±30 grams, and their average age was nine weeks old. The animals underwent acclimatization for seven days and were divided into five groups spaced in different rooms. Random numbers were generated using the standard function = RAND() in Microsoft Excel (Microsoft Corporation, Redmond, Washington, United States). They were kept in controlled environments with a 12-hour light/dark cycle, had their temperature regulated, and were fed and hydrated in the afternoon during this acclimatization phase. All animal care procedures adhere to ethical guidelines to minimize animal suffering. The rats were split into five randomly homogeneous groups, designated Groups A, B, C, D, and E, with three rats in each group. Group A: Ten percent of MUCASE was administered to three rats. Group B: A 20% dosage of MUCASE was administered to three rats. Group C: A 40 % dose of MUCASE was administered to three rats. Group D: As a positive control, three rats received Sofratulle® treatment. Group E: Three rats were given an HPMC basic formula as a negative control.

Before conducting the surgical procedure, the animals were anesthetized using inhaled ether. Subsequently, the dorsal area of the rat, designated for the procedure, was shaved using an electric clipper, moving from head to tail, and the area was adjusted to a size of 4 x 4 cm. The skin was then cleaned with cotton soaked in 70% alcohol. On the first day of the experiment, a 1.5 cm long and 0.2 cm wide surgical straight incision was created on the rat's back using a sharp surgical knife. Following the incision surgical, the splints were not applied to this minor incision wound size in rats due to several reasons: size and proportion, technical challenges, and animal welfare considerations; therefore, in this study, only topical treatment approaches are used, while splints are commonly used in wound management for larger wound animals or humans. Following the incision, the skin was cleaned with 0.9% saline, and a 2 × 2 cm bilayer, Sofratulle®, and Musa Cavendish's extract was directly applied onto the incision wound with gentle pressure to secure the layer in place over the wound site and to ensure uniform coverage and contact of the treatment with the wound surface while minimizing disruption to the healing process. The rats were divided into pre-established groups and dressed with sterile gauze and adhesive plaster. Every two days, the dressing was replaced until the wound healed.

Furthermore, exudation measurement, wound healing percentage, and contraction during seven days were the primary criteria assessed in this investigation. The measurements of exudation, bleeding, adhesive, and odor of the dressing were monitored by macroscopic examination and graded on a four-point scale of 0-low (0% - 25%), 1-moderate (26% - 50%), 2-high (51% - 75%), and 3-very high (76%-100%). Meanwhile, measuring wound closure or wound contraction, by taking pictures of the rats after the experiment and on the day of surgery; a ruler is positioned next to the wound site for proper scaling. Image software was used to measure the wound area at each point, and Microsoft Excel was used to enter the results [20,21]. Furthermore, according to previously established definitions, the percentage of wound healing was measured using millimeters from the wound edge, and the percentage of wound duration was calculated based on the amount of time required for healing from the day of incision with the following formula: wound healing time = (initial radius-final radius) [22]. The area of wound closure/wound contraction was calculated (%) with the following formula: % wound contraction = {(initial wound area − current wound area)/initial wound area} × 100 [23]. Wound area data are expressed as mean ± standard deviation.

Phytochemical composition of Musa Cavendish

In the pursuit of understanding the phytochemical composition of Musa Cavendish, several studies aimed to comparatively evaluate the content within its peel and stem, employing various extraction methods detailed in Table 2. This investigation sheds light on the diverse array of phytochemicals in the stem extract, encompassing compounds such as tannins, saponins, flavonoids, steroids/terpenoids, phenolics, catechin, and gallocatechin. The extraction results revealed a rich profile of phytochemicals, providing valuable insights into the botanical constituents of Musa Cavendish. By seamlessly integrating the details from Table 2, these findings illuminate the distinct characteristics of the stem's phytochemical composition, offering a comprehensive understanding of the plant's chemical constituents. This comparative analysis is a crucial step towards unraveling the wound healing potential applications and benefits associated with the diverse phytochemicals in Musa Cavendish.

**Table 2:** Phytochemical assessment of Musa Cavendish extract Examination locations a: Agricultural Technology Laboratory, Lampung State Polytechnic, 2022 [24]; b: São João do Polêsine (RS, Brazil) [25]; and c: Laboratory of Biostructural Chemistry, Graduate School of Life Sciences, Tohoku University, Japan [26]

Compound examination	Reagent	Description		Test location, a,b,c, and reference
		Banana peel	Banana stem	
Alkaloid	Meyer's test	(-)	(-)	[24]
	Dragendorff's test	(-)	(-)	[24]
Flavonoid	Bate Smith & Metcalf's method	(+)	(+)	[24]
	2,2-diphenyl-1-picrylhydrazyl, the ferric thiocyanate	(+)	(+)	[25]
Tannin/Polyphenol	(+)FeCl3	(+)	(+)	[24]
Saponin	Forth TEST	(+)	(+)	[24]
Steroid/Terpenoid	Concentrated HCL + Sulfuric acid	(+)	(+)	[24]
Catechin	Folin–Denis method	(+)	(+)	[26]
Phenolics	The ferric thiocyanate	(+)(+)	(+)	[26]
	2,2-diphenyl-1-picrylhydrazyl and the ferric thiocyanate	(+)	(+)	[25]
Gallocatechin	The ferric thiocyanate	(+)(+)	(+)	[26]

Analytical statistics

For every piece of quantitative data gathered, the arithmetic mean and standard deviation were calculated. It carefully measured the wound length throughout four intervention sessions spanning seven days. These measurements were then used to calculate the percentage of wound healing. To ensure data reliability, it performed the Shapiro-Wilk test to check for a normal distribution. Additionally, Levene's test was applied to confirm the consistency within each group and in cases where a significant difference was found through Levene's test, Welch's One-Way ANOVA was employed. If Welch's One-Way ANOVA revealed notable results, the Games Howell post-hoc test was conducted to identify specific groups that showed significant differences within the sample. These statistical analyses were conducted using IBM SPSS Statistics for Windows, Version 26 (Released 2019; IBM Corp., Armonk, New York, United States). It maintained a significance threshold of p < 0.05 throughout our analysis.

## Results

The quantitative data analysis, utilizing MUCASE at concentrations of 20%, 40%, and Sofratulle®, demonstrated in Table 3 a consistent reduction in overall exudation concerning incision wound interventions in comparison to MUCASE 10% and the negative control, achieving statistical significance (p < 0.05).

**Table 3:** Exudation parameters of incision lesions after topical treatment with MUCASE 10, MUCASE 20, MUCASE 40, Sofratulle and negative control (n = 15) in rats

Parameters	Groups	N	Weight/gr average	Days after wound Induction (scale (%± SD))			
				One day	Three days	Five days	Seven days
Exudation (volume, type, and odor)	MUCASE 10	3	161	1 (45.67 ±3.79)	1 (40.7± 2.1)	0 (27.3±1.5)	0 (8.7± 1.2)
	MUCASE 20	3	170	1 (48.33 ± 2.89)	0 (23±2.65)	0	0
	MUCASE 40	3	162	1 (42.33±2.52)	0 (20.33 ±2.52)	0	0
	Sofratulle	3	168	1 (46±3.61)	0 (20.67±3.79)	0	0
	Negative control	3	137	1 (46±3.61)	1 (34.33±4.04)	0 (28.67±3.06)	0 (16.67± 2.31)

**Figure 3:**
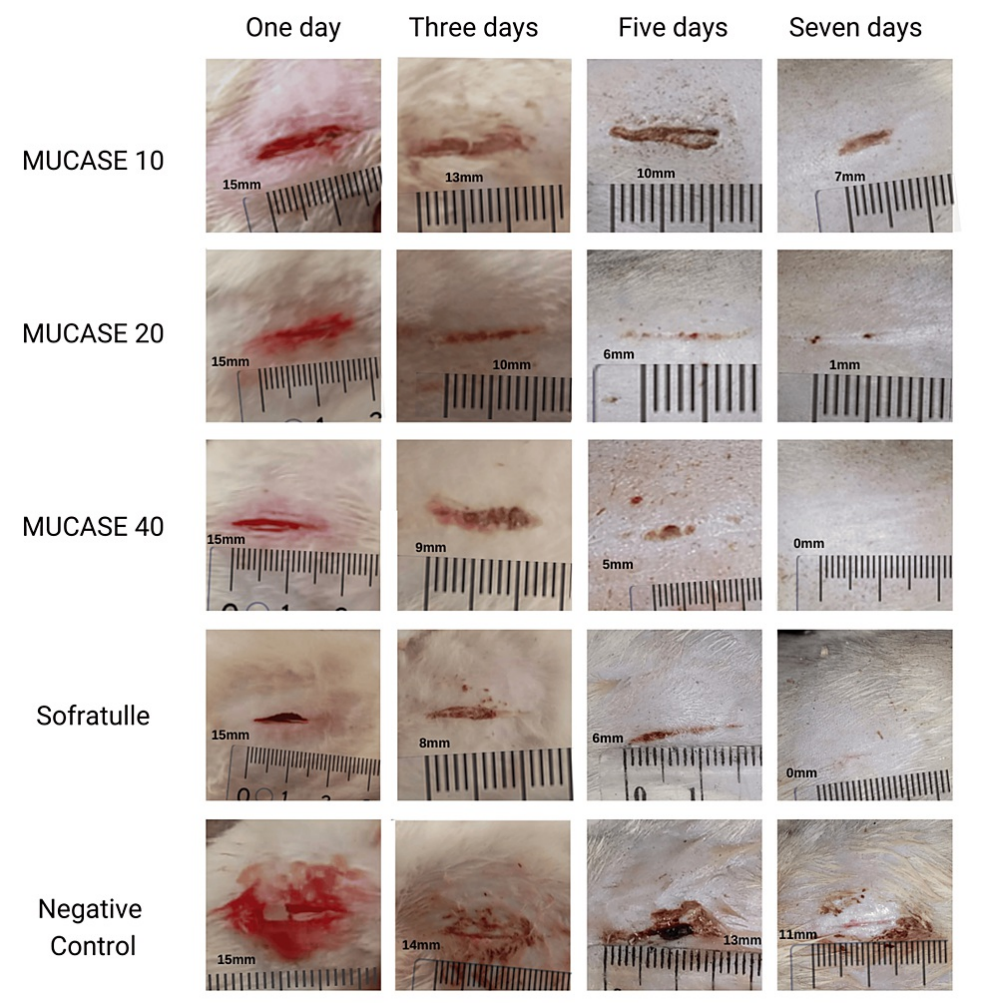
Arrangement of rat illustrations When the wounds were scabbing, the figures were measured by carefully observing the wound under good lighting to identify its boundaries and gently removing a portion of the scab if safe for wound length or contraction measurements. Then, using a ruler, document its observations and track its changes over time.

The peak of exudation was observed on the first day during the secondary dressing change, with a higher occurrence in areas treated with MUCASE 20%, 40%, and Sofratulle® (nine rats) compared to negative control and MUCASE 10% (six rats). Subsequently, a decline in exudation was noted as the healing progressed over the subsequent days (Figure 3).

Throughout the randomization-controlled research framework involving five pharmaceutical interventions over three days, no persistent bleeding occurred during the healing period. The frequency and duration of dressing changes exhibited no discernible difference within the confines of the study between wounds covered with MUCASE 20%, 40%, and Sofratulle®, MUCASE 10%, and wounds treated with the negative control.

The normality test results, indicating a value of p > 0.05, confirmed the normal distribution of the data. However, the Levene test revealed non-homogeneity in some data (p < 0.05). Subsequently, Welch's One-Way ANOVA was employed, revealing a significant mean difference among the treatment groups (p = 0.004 < 0.05). According to this statistical analysis, the application time of MUCASE 10%, 20%, 40%, Sofratulle®, and negative control dressings gradually decreased as the epithelialization process advanced, with the dressing completely absorbed after application. The primary dressing was changed every two days following the previous day's discharge, with no significant variation in the application time of the five dressings. On average, wound healing durations were 0.79 ± 6.70 days for MUCASE 40%, 20%, and Sofratulle®, 6.67 ± 8.31 days for MUCASE 10% and 13.67 ± 7.16 days for negative control. Wound closure was initiated on the second day and concluded by the sixth day. They indicated a notable disparity in the time required for wound healing, even in the latter stages of the process (Table 4).

**Table 4:** Following the intervention, the mean and standard deviation of wound surface area (WSA) with a 95% confidence interval for each group WSA: wound surface area ^a^Statistically significant via Welch's One-Way ANOVA <0.05

Mean, standard deviation, and p-value for WSA after the treatment (mm^2^)					p-value
	One day	Three days	Five days	Seven days	
Mucase 10%	15.67±0.577	14.33±1.155	12.33±1.155	6.67±2.309	0.013^ a^
Mucase 20%	15.67±0.577	12.67±0.577	7.00±1.000	1.03±0.950	0.000^ a^
Mucase 40%	15.33±0.577	10.33±0.577	4.33±0.577	0.33±0.577	0.000^ a^
Sofratulle^®^	15.33±0.577	10.67±1.155	5.00±1.000	1.00±1.000	0.000^ a^
Negative control	16.00±1.000	15.00±1.000	14.67±1.155	13.67±1.155	0.268

Examination of significant differences between certain pairs of groups among the five treatment groups revealed significant differences among specific groups (Table 5). A significant distinction was observed between the negative control group and the 20%, 40%, and Sofratulle® groups (p < 0.05). At the same time, no significant difference was identified between the negative control group and the 10% group (p > 0.05). Nonetheless, the MUCASE 40%, MUCASE 20%, and Sofratulle® groups exhibited no significant differences (p > 0.05). The differences between the control and treatment groups emerged from the second day of observation, remaining significant until the seventh day. On the third day, MUCASE 20%, MUCASE 40%, and Sofratulle® dressings outperformed negative control and MUCASE 10%. Despite varied recovery stages, the healing process commenced on the first day and continued until the fourth day. Rapid tissue formation occurred from the fifth to the seventh day, resulting in wound healing and hair regrowth. The results suggest that the three phases of wound healing in rats with incision wounds can be expedited using MUCASE as a topical dressing. Wound improvement became evident on the fifth day, with MUCASE 40% and Sofratulle® dressing groups exhibiting superior wound healing.

**Table 5:** Examination of significant differences between specific pairs of groups among the five treatment groups (MUCASE 10, 20, 40, Sofratulle®, and negative control) via Games Howell post hoc analysis

(I) Intervention level		Mean difference (I-J)	Std. error	Sig.	95% Confidence interval	
					Lower bound	Upper bound
MUCASE 10 %	MUCASE 20%	3.167	2.025	0.537	-2.93	9.26
	MUCASE 40%	4.667	2.047	0.195	-1.50	10.84
	Sofratulle®	4.250	1.989	0.246	-1.73	10.23
	Negative control	-2.583	1.154	0.224	-6.20	1.03
MUCASE 20%	MUCASE 10 %	-3.167	2.025	0.537	-9.26	2.93
	MUCASE 40%	1.500	2.427	0.971	-5.70	8.70
	Sofratulle®	1.083	2.379	0.991	-5.97	8.14
	Negative control	-5.750^*^	1.742	0.041	-11.30	-0.20
MUCASE 40%	MUCASE 10 %	-4.667	2.047	0.195	-10.84	1.50
	MUCASE 20%	-1.500	2.427	0.971	-8.70	5.70
	Sofratulle®	-0.417	2.398	1.000	-7.53	6.70
	Negative control	-7.250^*^	1.768	0.010	-12.89	-1.61
Sofratulle®	MUCASE 10 %	-4.250	1.989	0.246	-10.23	1.73
	MUCASE 20%	-1.083	2.379	0.991	-8.14	5.97
	MUCASE 40%	0.417	2.398	1.000	-6.70	7.53
	Negative control	-6.833^*^	1.700	0.012	-12.25	-1.42
Negative control	MUCASE 10 %	2.583	1.154	0.224	-1.03	6.20
	MUCASE 20%	5.750^*^	1.742	0.041	0.20	11.30
	MUCASE 40%	7.250^*^	1.768	0.010	1.61	12.89
	Sofratulle®	6.833^*^	1.700	0.012	1.42	12.25

**Figure 4:**
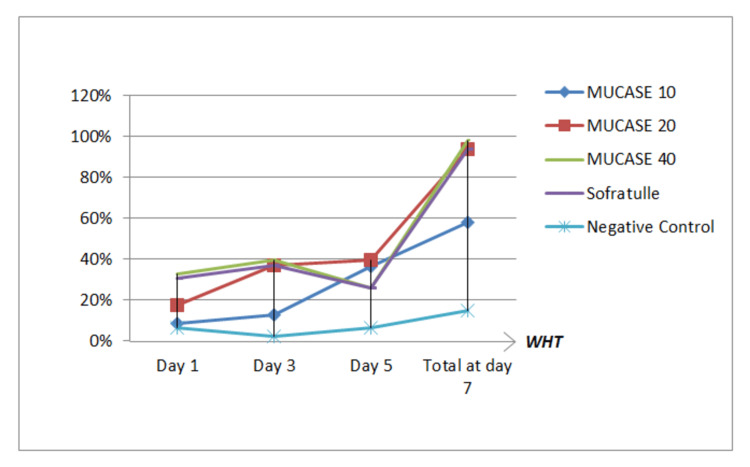
Illsutrates wound contraction after each group between days one and seven (n = 5) for the experimental groups (MUCASE 40%, 20%, 10%), Sofratulle®, and negative control. On the seventh day, there was no noticeable distinction between the Sofratulle®, MUCASE 40%, and 20% groups. However, significant differences were identified when comparing these groups with the MUCASE 10% and negative control groups. WHT: wound healing time

Wound contraction results over seven days, as depicted in Figure 4 and Table 6. It indicated the average percentage of wound contraction to the initial wound on days one, three, five, and seven as 19% ± 0.12%, 26% ± 0.17%, 27% ± 0.13%, and 71% ± 0.36%, respectively. On the first day, MUCASE 40% displayed a more significant contraction percentage (33%) compared to MUCASE 20% (17%) and MUCASE 10% (9%), despite subsequent contraction decrease. Nevertheless, a significant difference was observed on the first day when comparing MUCASE 40% and Sofratulle® (31%) with negative control (6%). Three days later, MUCASE 40%, MUCASE 20%, MUCASE 10%, Sofratulle® (37%), and negative control (2%) achieved maximum contractions of 33%, 39%, 36%, 37%, and 2%, respectively. Although MUCASE 40% exhibited the most significant contraction on the third day, no appreciable difference in wound contraction was observed between these groups on the fifth and seventh days. On the seventh day, distinct differences in the wound contraction of MUCASE 20% (58%) and negative control (15%) were noted, while MUCASE 40%, Sofratulle®, and MUCASE 20% demonstrated no significant alterations throughout the observation period. The wound area consistently diminished over time, with no appreciable variation in wound contraction among the three groups on the seventh day.

**Table 6:** Percentage wound contraction Each value is the mean ± SEM, standard versus n=15, SEM: standard error of mean

Postwounding days	Percentage wound contraction (mean ± SEM)				
	MUCASE 10	MUCASE 20	MUCASE 40	Sofratulle	Negative Control
1	15.67 ± 0.33	15.33 ± 0.33	15.33 ± 0.33	15.33 ± 0.33	16.00 ± 0.58
3	15.00 ± 0.50	14.00 ± 0.00	12.83 ± 0.33	13.00 ± 0.50	15.50 ± 0.58
5	13.33 ± 0.67	9.83 ± 0.44	7.33 ± 0.33	7.83 ± 0.60	14.83 ± 0.60
7	9.50 ± 1.00	4.00 ± 0.58	2.33 ± 0.17	3.00 ± 0.00	14.67 ± 0.67

## Discussion

Wound healing, tissue regeneration, and recovery from injury involve strategies such as wound contraction to expedite healing [20]. Recent studies highlight the potential of herbal or synthetic composites, including plant-derived materials, in enhancing wound healing efficiency [4-6]. However, limited scientific literature exists on the therapeutic effects of topical agents in skin wound healing [27]. Incision wounds demand careful attention to prevent infection and necessitate specialized treatment [20,28]. This study compares the impact of varying MUCASE concentrations on incision wound healing, revealing that MUCASE at 40%, 20%, and 10% accelerates healing compared to controls.

Incision wounds often penetrate deep dermal layers, affecting hair follicles and tissue glands [29]. Complete wound healing within six days was observed for MUCASE 40%, 20%, and Sofratulle®, contrasting with 6.67 ± 8.31 days for MUCASE 10% and 13.67 ± 7.16 days for negative control. Notably, 40% of MUCASE exhibited the most efficient healing. Different concentrations of MUCASE influenced wound contraction, aligned with its protective effects against oxidative damage and antibacterial, antifungal, and antioxidant properties [24-26,30].

With the increasing prevalence of antibiotic resistance, effectively treating wound infections has become a significant challenge in wound recovery [30]. In this context, using natural compounds such as MUCASE has proven promising in reducing exudation, potentially preventing infection, and as a natural wound healing agent. Flavonoids, for example, have been studied extensively for their wound-healing effects, including increasing collagen synthesis and angiogenesis and inhibiting microbial growth and inflammation [24,25]. Tannins/polyphenols have astringent properties that aid wound contraction and promote tissue repair [24]. Saponins exhibit antimicrobial and anti-inflammatory activity, contributing to wound sterilization and reduction of inflammation [24]. Steroids/terpenoids have been reported to accelerate wound healing by increasing fibroblast proliferation and collagen synthesis [24]. Catechins, phenolics, and gallocatechins are potent antioxidants that scavenge free radicals, thereby protecting cells from oxidative damage and promoting tissue regeneration [24-26].

Wound contraction, a vital healing aspect, was effectively enhanced by MUCASE 40%, 20%, and Sofratulle®. While MUCASE 40% demonstrated superior healing and early contraction, MUCASE 20% also proved effective. Scab formation and epithelial cell coverage in the MUCASE 40% group expedited healing, contrasting with prolonged scarring in the MUCASE 10% and negative control groups. Sofratulle® was deemed safe and effective, albeit costlier than MUCASE. Rivaling the efficacy of Sofratulle®, MUCASE, especially at 40% and 20%, emerges as a cost-effective and potent incision wound care dressing.

Treating incision wounds with Sofratulle® is safe and effective, albeit more expensive than MUCASE dressings. Cost-effectiveness studies reveal that reasonably priced topical medicines are economically superior to Sofratulle® [14]. In providing efficient and reasonably priced incision wound care, MUCASE, especially at concentrations of 20% and 40%, emerges as a potential competitor to Sofratulle®. The study's focus on minor incision wounds limits the exploration of hyperpigmentation and hypertrophic scarring, common in larger burn wounds treated with Sofratulle®.

Several limitations need to be considered in this study. Firstly, the relatively small size of the research groups and using rats as a model may limit the generalization of these findings to the human population. Therefore, multi-center studies involving more extensive human samples are required to validate and support our research findings. Second, the straight incision wound depth was only evaluated, not including the skin tension; therefore, future research can benefit from using objective assessment instruments like the laser Doppler and tensiometer with circle wound or another type of incision and excision wound, which can improve accuracy and group comparison. Following this, due to the minor incision wound size in rats then, the splints were not applied due to several reasons: size and proportion, technical challenges, and animal welfare considerations; therefore, in this study, only alternative approaches are used, while splints are commonly used in wound management for larger wound animals or humans. Additionally, although wound areas were randomly selected, there is a possibility of variation in incision wound depth around the rat's back, which could affect the results. This study also did not include a long-term evaluation, so differences in scar quality cannot be discussed in detail.

## Conclusions

In conclusion, the study addressed the need for innovative wound healing strategies, emphasizing plant extracts, mainly from Musa Cavendish banana stems. The study demonstrated that MUCASE, especially at concentrations of 40% and 20%, accelerates wound healing, reduces exudation, and enhances wound contraction in incision wounds compared to lower concentrations and a negative control group. To facilitate efficient and swift wound healing, MUCASE concentrations exceeding 20% and Sofratulle® are suitable as wound dressings. Although both options exhibit advantages, MUCASE could be a cost-effective and potent alternative to commercial wound dressings, offering potential benefits in antibacterial, anti-inflammatory, and antioxidant properties. Despite some limitations, this study contributes valuable insights into the application of plant extracts in wound care, emphasizing the need for further research to establish MUCASE's efficacy in larger-scale and long-term clinical settings.
